# Opportunities and considerations for visualising neuroimaging data on very large displays

**DOI:** 10.12688/f1000research.9522.1

**Published:** 2016-09-02

**Authors:** Matthew B. Wall, David Birch, May Y. Yong

**Affiliations:** 1 Imanova Centre for Imaging Sciences, London, W12 0NN, UK; 2 Division of Brain Sciences, Imperial College London, London, SW7 2AZ, UK; 3 Clinical Psychopharmacology Unit, University College London, London, WC1E 7HB, UK; 4 Data Science Institute, Imperial College London, London, SW7 2AZ, UK

**Keywords:** Neuroimaging, fMRI, PET, visualisation, data observatory, display technology, eye-tracking, multiple sclerosis.

## Abstract

Neuroimaging experiments can generate impressive volumes of data and many images of the results. This is particularly true of multi-modal imaging studies that use more than one imaging technique, or when imaging is combined with other assessments. A challenge for these studies is appropriate visualisation of results in order to drive insights and guide accurate interpretations. Next-generation visualisation technology therefore has much to offer the neuroimaging community. One example is the Imperial College London Data Observatory; a high-resolution (132 megapixel) arrangement of 64 monitors, arranged in a 313 degree arc, with a 6 metre diameter, powered by 32 rendering nodes. This system has the potential for high-resolution, large-scale display of disparate data types in a space designed to promote collaborative discussion by multiple researchers and/or clinicians. Opportunities for the use of the Data Observatory are discussed, with particular reference to applications in Multiple Sclerosis (MS) research and clinical practice. Technical issues and current work designed to optimise the use of the Data Observatory for neuroimaging are also discussed, as well as possible future research that could be enabled by the use of the system in combination with eye-tracking technology.

## Introduction

A natural trend in many scientific disciplines is towards greater size and complexity of the empirical data sets that are collected. This may be driven by the development of entirely new research methodologies or diagnostic tests, further refinements of existing technology ( *e.g.* greater resolution of imaging, or higher speed of sampling), or by the incorporation of multiple measurement methods to examine a single question. In the era of ‘Big Data’ (Katal *et al.*, 2015; Marx, 2013) some scientists are now developing specialist techniques to handle truly enormous data sets.

This trend is certainly evident in the field of neuroimaging. Functional Magnetic Resonance Imaging (fMRI) is now the workhorse method in cognitive neuroscience and can generate impressively large and complex data sets. Recent advances in fMRI acquisition software to achieve increased spatial and temporal resolution ( *e.g.* Moeller *et al.*, 2010) have driven a further increase in data volumes. Large scale endeavours such as the Human Connectome Project (HCP; Van Essen *et al.*, 2013) aim to gather a variety of different data from large cohorts. The HCP is currently acquiring data using four different MRI procedures (structural, resting-state fMRI, task fMRI, and diffusion imaging), from 1200 subjects, with a sub-set also completing magnetoencephalography (MEG) and electroencephalography (EEG) scans, and a further sub-set also completing additional scans on a high-field strength (7 Tesla) MRI scanner. With additional demographic, behavioural, and questionnaire measures, the final HCP data set will be a tremendous resource, but its sheer size will require specialist methods of data-handling and analysis.

The HCP illustrates two common features of modern neuroimaging research. First is the collection of multiple types of data from a set of subjects using a single imaging modality, most commonly MRI. These may include task fMRI, resting-state fMRI, diffusion MRI, Arterial Spin Labelling (ASL), Magnetic Resonance Spectroscopy (MRS), or a number of other specialist techniques. The second is the advent of true multi-modal neuroimaging research, where combinations of two (or more) methods are used, either simultaneously or independently. Combined fMRI-EEG studies ( Huster *et al.*, 2012) combine the high spatial resolution of MRI with the high temporal resolution of EEG, often with simultaneous acquisition. MRI and MEG have also been used successfully ( *e.g.* Carhart-Harris *et al.*, 2016) and provide similarly complementary data, though not simultaneously. MRI and Positron Emission Tomography (PET) data can be collected independently ( *e.g.* Colasanti *et al.*, 2016; Rabiner *et al.*, 2011) or simultaneously (using the new generation of combined PET/MR scanners; Bailey *et al.*, 2015) and combine PET-derived information on neurochemistry with structural or functional MRI measures. Multimodal imaging has also begun to filter through into clinical practice with some diagnostic criteria now incorporating imaging markers of neurodegenerative disorders ( *e.g.* in Multiple Sclerosis; Polman *et al.*, 2011).

These multi-paradigm and multi-modality studies are of great value in providing complementary and converging evidence to characterise healthy brain function, examine various disease states, and in drug development ( Matthews *et al.*, 2011). The challenges involved in analysing and manipulating large multi-modal datasets have been partly addressed by advances in hardware and software. For example, the issue of fusing images from different modalities has largely been solved by modern software ( *e.g.* Gunn *et al.*, 2016) using automated co-registration algorithms that generally produce good-quality results. One remaining challenge is the provision of appropriate visualisation technologies that can provide an overview of a set of (sometimes disparate) results images, and can enable accurate interpretations to be made. Many specialised software tools now exist for visualising neuroimaging data (a comprehensive list, and a useful guide to visualisation can be found in Madan, 2015) however, their utility is necessarily constrained by the users display hardware; typically a single, or several standard desktop computer monitors. Advances in display technology have only been incompletely addressed, with most tools not optimised for larger displays, and also not incorporating modern user-interface features such as touch input. This occurs for two reasons, firstly that physical display hardware has only recently begun to support the higher resolutions required to display the higher fidelity data which are now routinely captured. Secondly the software used to display scientific data has not benefited from the revolution in distributed, cloud computing which data processing systems such as Map-Reduce and Hadoop provide ( Patel *et al.*, 2012).

**Figure 1.**
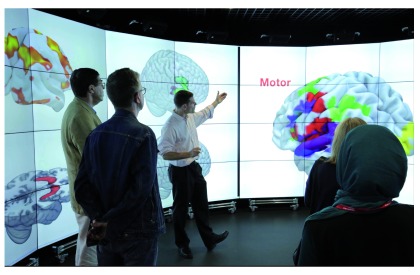
Neuroimaging data presented on the Data Observatory. Professor Oliver Howes presenting multimodal imaging data at the MRC Clinical Sciences Centre's "Hearts and Minds" public engagement event, 23 June 2016. Photo Credit: Susan Watts, MRC Clinical Sciences Centre, Imperial College London, reproduced with permission.

**Figure 2.**
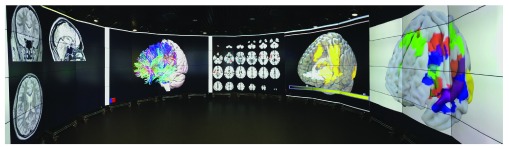
Panoramic image of the Data Observatory. A panoramic image of the Data Observatory, with all five sections displaying a different neuroimaging modality and/or visualisation type. Photo credit: Authors MW and DB.

To address these challenges and enable high-resolution collaborative exploration of detailed scientific data a new generation of advanced visualisation suites are being developed ( Febretti *et al.*, 2013). One example is the KPMG Data Observatory (DO) at Imperial College London. This is a panoramic display covering a 313 degree arc with a 6 m diameter, providing an immersive and collaborative space for exploration of data (see Figure 1 and Figure 2). The key differentiator of the space is its high resolution which totals 132 megapixels, in contrast with the low-resolution projector based approach of traditional CAVE systems. The system is driven by 32 rendering nodes that enable distributed analysis and rendering of data, and the display area can be flexibly configured into either a single display surface, or a number of sections displaying different information sources or applications. The key goal of the observatory is to provide a collaborative space for research teams to explore and discuss data in a visual format.

This collaborative, high-resolution approach to visualisation has much to offer the neuroimaging community. Particularly:

1) The ability to view images at full resolution without the need for interruptive actions such as zooming or panning through an image.
2) The ability for multiple practitioners to share the same, high resolution, view of data for discussion in a collaborative environment.
3) The ability to simultaneously show many views of the same or complementary data; large-scale visualisation allows complementary data to be shown simultaneously and accessed by a turn of the head, which enables easy comparison.

These benefits are of particular value to collaborative interdisciplinary groups exploring such multi-modal imaging studies. One case study under exploration at Imperial College involves Multiple Sclerosis.

## Case study: Multiple sclerosis (MS)

MS is an autoimmune disease that affects more than 100,000 people in the United Kingdom alone ( Mackenzie *et al.,* 2014). It has a debilitating effect on various body functions including vision, motor and cognition; while there are treatment options available there is currently no known cure. MS assessments are made using objective clinical criteria, supplemented by findings of lesions in the central nervous system that are detectable on MRI scans over a period of time and space ( Polman *et al.*, 2011). T1 images, T2 images, and contrast-enhanced MRI using gadolinium are all useful techniques in this regard (Bakshi *et al.*, 2008). Other diagnostic tests include assessment of visual function (as visual deterioration occurs in over 80% of patients) using a Low-Contrast Sloan Letters Chart ( Baier *et al.*, 2005). Optical Coherence Tomography (OCT; Petzold *et al.*, 2010) or Visual Evoked Potentials (VEP; Schlaeger *et al.*, 2014) can also provide measures of retinal integrity and central nerve damage, respectively. Finally, functional tests and questionnaires can register cognitive and functional impairments. There are currently no known specific blood or cerebrospinal fluid (CSF)-borne biomarkers for MS ( Polman *et al.*, 2011), so diagnosis depends on a combination of these measures, and the relationship between these tests and disease progression (particularly in a predictive sense, *e.g.* Neema *et al.*, 2009) is an area of active research.

Synthesising and visualising the results of these varied tests is a challenge that is being addressed by current work on the DO. Lesion volume change and brain volume change data from analysis of MRI images are a critical components for tracking disease progression. Images from each contrast type (T1, T2, gadolinium-enhanced) provide unique information along with complementary limitations. The ability to register and view all modalities simultaneously enables the viewer to crosscheck the same regions of interest across large screens without the current need to toggle between screens or windows.

In addition, tools can be built to replicate inputs across imaging modalities, and between image sessions. A tool that highlights a region of interest on one modality can automatically replicate the marking of the same region across other modalities, on other sections of the DO display. Similarly, a lesion may be marked in the baseline image and have that mark replicated in a registered image from a follow-up session. The ability to view changes in these images simultaneously in the context of data collected from other tests such as OCT, VEP, functional criteria or radiological reports enables viewing of disparate sources of information in tight context.

The large display-area provides space to fit a timeline that incorporates imaging data, clinical events, treatments, written reports, and clinical test results; this gives a unique visualisation of the cause and effects of disease progression, treatments, and relapse events in MS. The flexibility and size of the display space enables novel visualisations, such as the scope to concurrently view individual results from a group of research subjects, or to view multiple sets of longitudinal data from a single subject. Clinicians and researchers can view, correlate, and cross-validate findings across heterogeneous data types within a single environment. As it is designed to be a collaborative environment for exploring images, multiple clinicians can highlight and share findings from different modalities and sources.

Environments such as the DO may one day become commonplace, however currently they are an expensive rarity, with only a few comparable systems existing worldwide ( *e.g.* the ‘ HIPerWall’ at University of California, San Diego). This currently strongly limits their accessibility to many researchers and clinicians. These constraints make the use of a tool such as the DO in current clinical practice impractical. The DO is more effectively deployed for clinical research purposes, or perhaps in consultant meetings, when high-level discussion of an individual case is required.

## Technical considerations

From a technical perspective, software used within such high-resolution environments must be adapted to cope with higher pixel densities and to work across a network of rendering computers. This is rarely a straightforward change, although with the advent of new rendering systems it is becoming easier. In general, vector-based graphics systems that display neuroimaging data as a 3D mesh using rendering engines like OpenGL (e.g. Surf Ice) work better than bitmap-based display tools, that are limited by the (often poor) underlying resolution of the images themselves. Ideally, display software needs to evolve to support distributed visualisation systems able to support display across a large rendering surface, and scalability to support high-resolution environments. Also important will be the development of appropriate algorithms to support decision-making, for instance to highlight areas of potential interest to clinicians for review. This method of focussing attention and insight will be a critical area of development in the near future, and will need to have extremely high levels of robustness and reliability, particularly if algorithms will eventually have some input into clinical decision-making. Machine-learning platforms such as Google’s Tensorflow ( Abadi *et al.*, 2016) are likely to be important components of such systems.

One potential area of investigation enabled by the DO is the quantification of how images are used within a visualisation space, particularly which data and which image regions are of most interest to clinicians. The key means to doing this is via head- and eye-tracking systems, which are starting to become available within such visualisation spaces. This would provide a means of identifying patterns of behaviour in how clinicians use images to identify the most salient features. One hypothesis worthy of further investigation is to explore how clinicians with different levels of experience explore, manipulate, and interpret a set of different images. Eye tracking can also be used to improve user experience, to ensure that the most commonly accessed information is placed in prominent display areas.

## Conclusions

Visualisation spaces such as the DO are relatively novel environments, and discovering the most effective ways of using them is still an on-going process. High-resolution spaces like the DO offer greater fidelity over previous large-scale systems, which can potentially drive greater insights. The large size of the space enables easy comparison and synthesis of multiple types of data, most obviously imaging formats, but also other clinical or research data types. Finally, the immersive collaboration space it provides can help to initiate and strengthen multi-disciplinary collaboration between clinicians, researchers, and data scientists. Large format displays like the DO have much to offer and will likely form an important part of future research and clinical practice.
