# Supplementary material for: Opportunities and considerations for visualising neuroimaging data on very large displays
Source: F1000Res. 2016 Sep 2;5:2157. [Version 1] doi: 10.12688/f1000research.9522.1 (PMC5031127; doi:10.12688/f1000research.9522.1)
Supplement: Peer review history

# Peer review: Referee response for version 1

**DOI:** 10.5256/f1000research.10257.r16066

**Published:** 2016-09-19

**Authors:** Joshua Balsters

**Affiliations:** 1 Neural Control of Movement Laboratory, Department of Health Sciences and Technology, ETH Zurich, Zurich, Switzerland

In this manuscript Wall *et al.* introduce the future potential of large display systems for visualizing neuroimaging data, making particular reference to their relevance for clinical neuroimaging by visualising multiple sources of neuroimaging information in parallel. I found this manuscript to be timely and well written. The MS case study also provides a clear example of how these tools can be used in real life.

 I whole-heartedly recommend this article is indexed after a couple minor grammatical errors are addressed.    

1. Third paragraph of the introduction – I think you mean to say “MRI and MEG have also been ‘fused’ …” rather than ‘used’. 
2. Pg 5 paragraph starting “Synthesising and visualising…” – change “are a critical components” to “are critical components”.

I have read this submission. I believe that I have an appropriate level of expertise to confirm that it is of an acceptable scientific standard.

# Peer review: Referee response for version 1

**DOI:** 10.5256/f1000research.10257.r16063

**Published:** 2016-09-08

**Authors:** Jens Foell

**Affiliations:** 1 Department of Psychology, Florida State University, Tallahassee, FL, USA

This manuscript describes a novel way to display large-scale data, including (but not limited to) data with a clear relation to neuroinformatics, with the goal of increasing the utility of complex multi-modal data. This is a relevant topic for neuroinformatics and neuroimaging, as it ensures that data accessibility advances in the same pace as the possibilities of data acquisition, data analysis, and data storage do. This manuscript is well written and provides a clear and comprehensive overview of the capabilities and logistics of one particular display system which is suitable to investigate functional or structural neuroimaging data and to compare changes over time.

 For these reasons, this manuscript will be relevant and interesting to the readership of F1000Research and I recommend its indexing after some minor technical points have been addressed (see below).

 Minor recommended changes:

 1) The very first citation ("Katal *et al.*, 2015"; paragraph 1, line 4) does not contain a hyperlink. The same is true for the citation "Bakshi *et al.*, 2008" in the first paragraph of the Case Study section.

 2) The abbreviation CAVE should be explained at its first instance in the text (5th paragraph)

I have read this submission. I believe that I have an appropriate level of expertise to confirm that it is of an acceptable scientific standard.
